# Cognitive improvement following repair of a basal encephalocele

**DOI:** 10.1007/s00701-017-3422-7

**Published:** 2017-12-17

**Authors:** Isabel Tulloch, Siobhan Palmer, Richard Scott, Dora Lozsadi, Andrew J. Martin

**Affiliations:** 1 Neurosurgical Department, Atkinson Morley Wing, St George’s Hospital, London, UK; 2 Department Clinical Health and Neuropsychology, St George’s Hospital, London, UK; 3 Neurology Department, St George’s Hospital, London, UK

**Keywords:** Memory impairment, Cognitive impairment, Temporal lobe, Encephalocele, Sphenoid sinus

## Abstract

We report the case of a 55-year-old woman presenting with progressive memory impairment secondary to a transsphenoidal encephalocele involving her dominant medial temporal lobe. Her clinical deterioration was accompanied by radiological progression in the encephalocele's size and associated encephalomalacia. Through a temporal craniotomy, her encephalocele was resected and the defect closed. Baseline neuropsychological assessment indicated global cognitive impairment, but post-operatively, she reported improved memory and concentration. Standardized assessment reflected an improvement in perceptual skills and an associated improved recall of a complex figure. This is the first case report to date of a patient's memory improving following treatment of a basal encephalocele.

## Introduction

An encephalocele is a pathological extension of the brain parenchyma into an osseous-dural defect in the skull base or cranial vault. Approximately 1.5% of encephaloceles are located within the skull base and are accordingly referred to as ‘basal’ [5]. We describe the rare case of a patient presenting with progressive cognitive impairment secondary to a basal encephalocele.

## Case Report

In 2015, a 55-year-old right-handed woman presented with 2 years of progressive attentional and prospective memory slips. She reported excessive fatigue, poor concentration, and difficulty maintaining conversations, struggled to remember recent events, names and learn new faces, and frequently forgot instructions at work. At home, she misplaced items and found it difficult to cook independently. She did not exhibit any indication of mood disorder. She also described a long-standing dripping sensation from her left nostril.

In 2005, she had been diagnosed with epilepsy manifest in complex partial and secondary generalized seizures with clinical and EEG findings supporting a right-sided temporal focus with no contralateral abnormalities. Her MRI brain at that time showed a small left-sided temporal encephalocele, without any evidence of a causative structural lesion on the right to explain these seizures. At the time of her presentation in 2015, she had not experienced any seizures since 2005 and continued on carbamazepine 800 mg daily. There was no history of trauma, infection, or endocrine symptoms, and no suggestion that she had ever had idiopathic intracranial hypertension.

On examination, she had a raised body mass index (BMI), and was neurologically intact with no rhinorrhea on provocation.

**Fig. 1:**
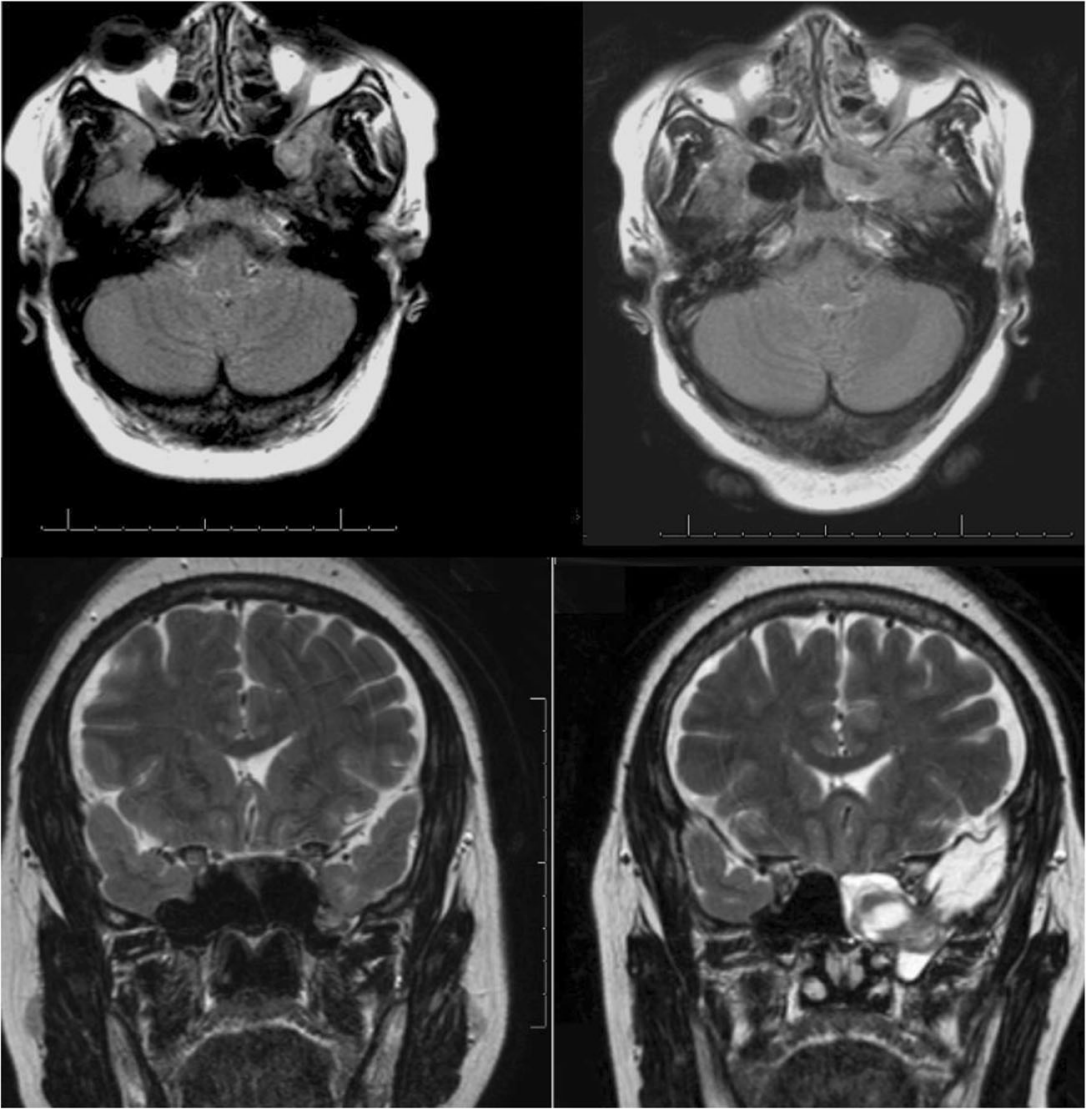
T2-weighted MRI of the brain. The MRI on the* left* is from 2005, and the one on the* right* is from 2015. They show the interval enlargement left-sided basal transsphenoidal encephalocele with associated encephalomalacia within the temporal lobe

MRI (Fig. 1) revealed a large transsphenoidal encephalocele with herniation of her dominant medial temporal lobe, and an ‘empty sella’. The encephalocele had enlarged notably over 10 years since her previous MRI and now caused ‘down traction’ on the temporal lobe.

**Fig. 2:**
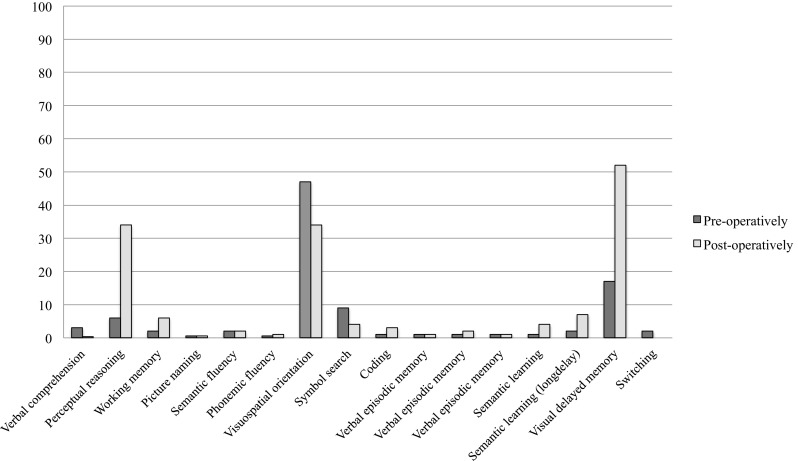
Pre- and post-operative neuropsychological assessment percentile results

Neuropsychological assessment was performed using the Wechsler Adult Intelligence Scale (WAIS-IV) [14]. The results of her pre-operative neuropsychological assessment are shown in Fig. 2. This assessment identified significant language and memory impairment consistent with dominant temporal lobe pathology, which could not be explained as secondary to translation, or by mood or environmental circumstances. Her perceptual reasoning and delayed recall of a complex figure were impaired, falling within the 6th and the 10–25th centile, respectively. She could not retain verbal information beyond one sentence with multiple intrusion errors at recall. Her verbal episodic memory and verbal recognition were both notably impaired, falling within the 1st and below the 2nd centile, respectively, indicating that she had a significant deficit in encoding verbal information into memory. She in part compensated for this using her visual memory, with her showing a limited ability to encode and retain visual information beyond a short delay.

Given her progressive cognitive decline, she underwent surgical repair of the encephalocele. A transcranial repair was performed as the defect was too laterally located to allow sufficient trans-nasal endoscopic access. A temporal craniotomy was performed extending to the floor and tip of the middle cranial fossa. Following durotomy, CSF was released from a lumbar drain to aid brain relaxation, and several adhesions were divided to reveal the lateral edge of the encephalocele extending through a well-defined rounded dural defect. The protruding brain was transected and the dural margin defined. The encephalocele was then debulked and the defect repaired with a free fat graft placed into the encephalocele cavity, intradural fascia lata, and fibrin sealant (Tisseel®). A free intradural fat graft was then sited and further Tisseel® applied. Following routine closure, lumbar drainage was continued for several days.

**Fig. 3:**
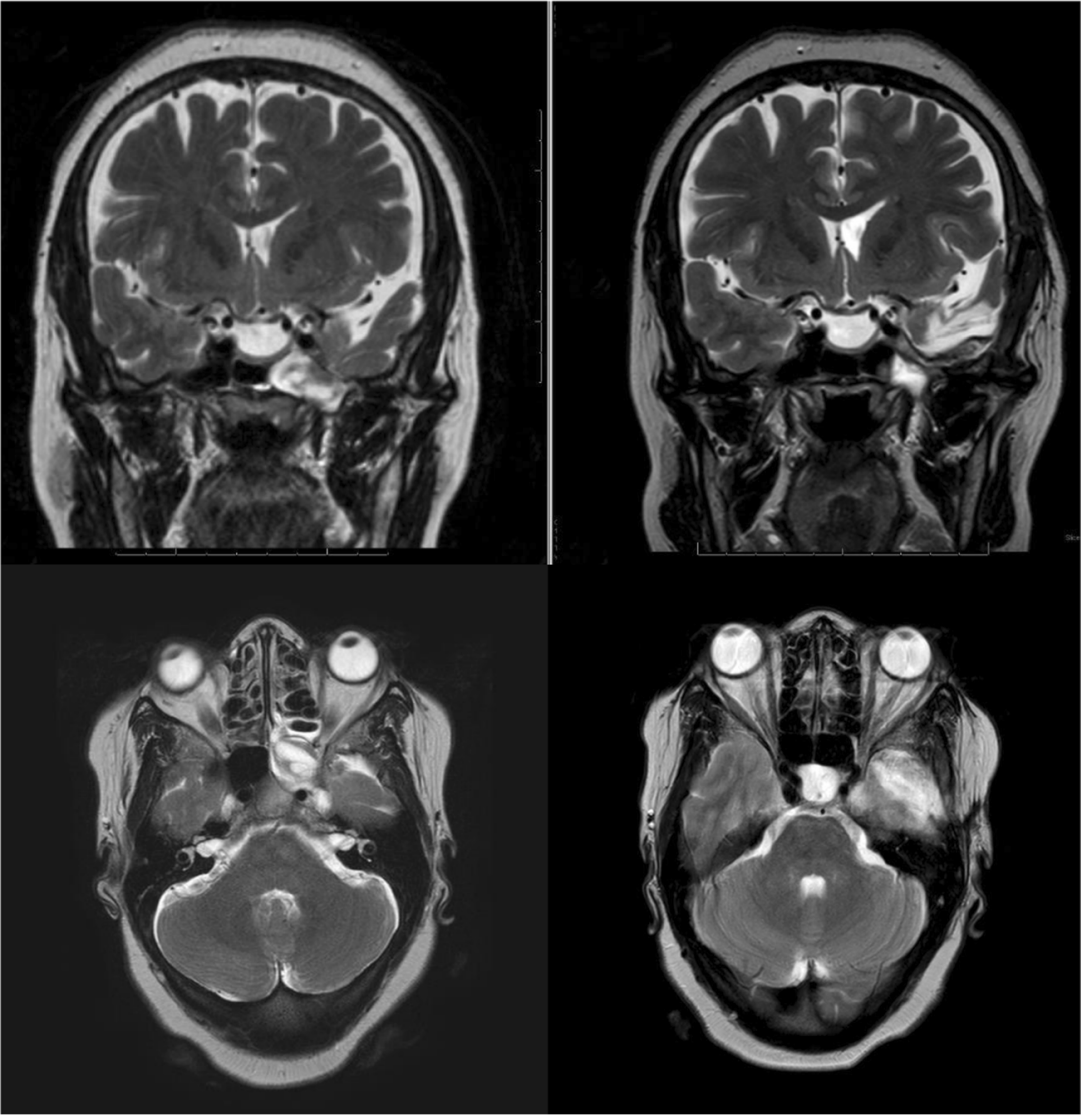
T2-weighted MRI of the brain. Her pre-operative imaging is on the* left*. On the* right*, her post-operative imaging is shown. The dysplastic temporal pole has been resected and a plaque of fatty material is visible within the encephalocele's former cavity

She made an excellent recovery with immediate resolution of her rhinorrhea. Post-operative MRI (Fig. 3) at 3 months showed a successful repair with relief of the ‘down traction’ on the temporal lobe.

Twelve months post-operatively, a second neuropsychological evaluation identified improvement in perceptual reasoning and visual delayed memory (Fig. 2). Perceptual Reasoning (WAIS-IV, Perceptual Reasoning Index) improved from the 6th to the 34th percentile and delayed recall of a complex figure improved from 10–25th percentile to the 50–54th percentile. However, her verbal episodic memory and verbal recognition both remained notably impaired. Subjectively, the patient and her family felt that she had steadily improved post-operatively. She no longer struggled to concentrate or complete household chores and work duties, and found conversations easier to follow. Fatigue and sleepiness was no longer reported as a concern. Overall, she was very satisfied with her surgical outcome.

## Discussion

Historically, basal encephaloceles are classified in accordance with their location into transsphenoidal, spheno-orbital, spheno-ethmoidal, spheno-maxillary, and transethmoidal subtypes [9]. Our patient had a left-sided transsphenoidal encephalocele (TSE).

The vast majority of TSEs are congenital, frequently being associated with craniofacial midline defects with their origin relating to the complex ontogeny of the sphenoid bone. Less commonly they are acquired, for example secondary to infection, surgery, trauma, malignancy, or elevated intracranial pressure (ICP) [12]. Our patient did not have any congenital craniofacial anomalies or any known precipitant. However, her pre-operative MRI had shown that her dominant temporal lobe potentially had ‘down traction’ forces upon it. This finding could be deemed to be suggestive of an elevated ICP, with the elevated pressure resulting in the downward displacement of the lobe through the osseous-dural defect. However, normal physiological variations in ICP also can contribute to this process. Importantly, our patient did not have any symptoms or signs consistent with an elevated ICP, nor did she have any other radiological features to support this diagnosis, rendering it an unlikely explanation for her encephalocele. We therefore have concluded that her encephalocele was most likely either congenital or spontaneous.

In 2013, Carlson et al. [2] evaluated 86 cases of surgically treated middle fossa encephaloceles. The characteristics of these patients bore great similarity to our patient, with the average age at the time of surgery being 52.3 years, almost 50% having an ‘empty sella’ and spontaneous encephaloceles being more common in females, particularly those with elevated BMIs.

The precipitant for our patient's review, investigation, and subsequent surgery was her report of progressive cognitive impairment. This has not previously been recognized as a presenting symptom of basal encephaloceles [13].

Our patient's encephalocele involved the medial aspect of her dominant temporal lobe. This part of the brain plays a critical role in memory. The central component to the medial temporal lobe memory system is the hippocampal formation. Its structural integrity is essential for both declarative memory and memory consolidation [7]. Hence, the progressive medial temporal changes noted on her interval imaging were likely to be a major contributor to her memory impairment.

The standard indications for surgical treatment of TSEs include persistent rhinorrhea, intracranial infection, epipharyngeal respiratory distress, and progressive neurological deficits. Surgery is aimed at closing the defect. Generally, the herniated tissue is resected without causing morbidity [6]. Surgical techniques are broadly separated into endoscopic and open surgical approaches. TSEs can be particularly difficult to control through an endoscopic approach due to an associated lack of control of the lateral recess of the sinus [3]. Cases are assessed individually, taking into account the encephalocele's location, shape, and size.

We performed a small temporal craniotomy, providing an excellent approach to the defect. Several authors have utilized this technique to access similarly located encephaloceles [3, 1]. Alternative approaches have also been reported; including pterional and orbitozygomatic approaches generating mixed results [9, 10].

The general consensus is that a multilayer graft is required to close the dural defect. Savva et al. [11] reviewed 52 transcranial approaches to post-operative dural defects. They found that multilayer defect closure techniques were most successful, particularly those combining allogenic materials with free autologous grafts. Carlson et al. also supported this conclusion, advocating an autologous multilayer repair [2]. We used a multilayer dural closure technique.

We used a lumbar drain both intra-operatively to facilitate brain relaxation and post-operatively to augment the union of the graft. However, in the absence of elevated ICP, the use of a lumbar drain can be deemed prophylactic. Several authors have also reported successful closures following the use of a drain prophylactically to enhance the integrity of their repair [4, 8].

Our patient reported two positive outcomes post-operatively. First, she noted complete resolution of her rhinorrhea. Second, over time she and her family felt that her memory and concentration notably improved. Neuropsychological assessment identified an improvement in her perceptual reasoning, and there was also an improvement in her visual delayed memory secondary to change in her perceptual skills. Her post-operative MR imaging was satisfactory and her symptoms have not recurred.

## Conclusion

This is the first report of a patient with this type of encephalocele requiring surgical input due to progressive memory impairment. It is also the first report of a significant improvement in cognitive functioning being noted after this type of surgery. As an isolated report, the extrapolations from this case are limited. However, this case is important not only to fellow surgeons who are confronted with similar encephaloceles and have to weigh the risks and benefits of surgery, but it is also anecdotally valuable for clinicians who assess patients with memory disorders who may not be aware of this potential, albeit rare, diagnosis.
